# Pediatric Encephalopathy and Complex Febrile Seizures

**DOI:** 10.1177/00099228221084422

**Published:** 2022-03-30

**Authors:** Amanda Yaworski, Rashid Alobaidi, Natarie Liu, Janette Mailo, Janani Kassiri

**Affiliations:** 1 Division of Neurology, Department of Pediatrics, Faculty of Medicine and Dentistry, University of Alberta and Stollery Children’s Hospital, Edmonton, AB, Canada; 2 Division of Pediatric Critical Care, Department of Pediatrics, Faculty of Medicine and Dentistry, University of Alberta and Stollery Children’s Hospital, Edmonton, AB, Canada

## Case Report

Three pediatric patients presenting with febrile seizures, ultimately diagnosed with a rare encephalopathy, are discussed. The patients’ families provided informed consent for case publication and the IRB-approved data collection (Pro00099091).

**Figure 1.**
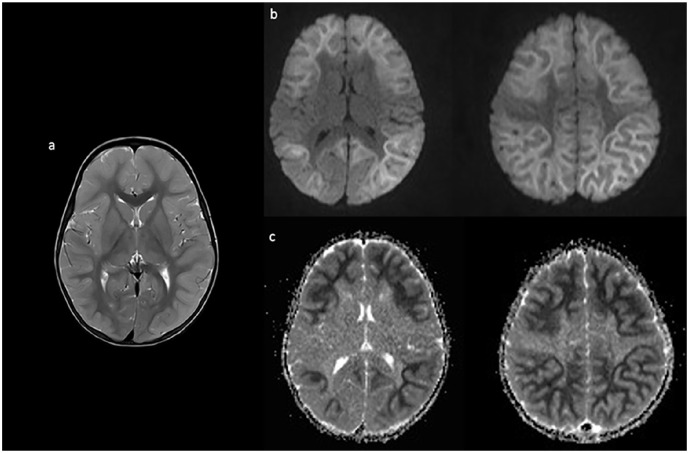
Axial T2 imaging on day 6 of illness shows cortical thickening and increased T2 signal (a) as well as diffusion restriction (b) and corresponding apparent diffusion coefficient (ADC) changes (c) in a bright tree appearance, sparing the rolandic area.

*Case 1*: A 22-month-old, developmentally normal white female, with a history of a single febrile seizure, presented with a prolonged (10-20 minutes) seizure in the context of a fever on the third day of a respiratory illness. The peripheral white blood cell count and C-reactive protein (CRP) were elevated at presentation, while head computed tomographic (CT) and cerebrospinal fluid (CSF) investigations, including an encephalitis panel, were normal. On day 4, her alanine transaminase (ALT) became elevated (>1200 U/L) but her INR and ammonia levels remained normal. Her sputum was positive for enterovirus but infectious disease screening was otherwise negative. Electroencephalography (EEG) showed diffuse generalized slowing, intermittent rhythmic delta activity, intermittent multifocal sharp waves, and an absence of normal sleep features in keeping with diffuse cerebral dysfunction; brain magnetic resonance imaging (MRI) was normal. On day 5, she had a seizure without fever but with further increase in liver enzymes. On day 6, a repeat brain MRI (Figure 1) showed diffusion restriction with a bright tree appearance (BTA) throughout the supratentorial cortices with sparing of the peri-rolandic areas.

**Figure 2.**
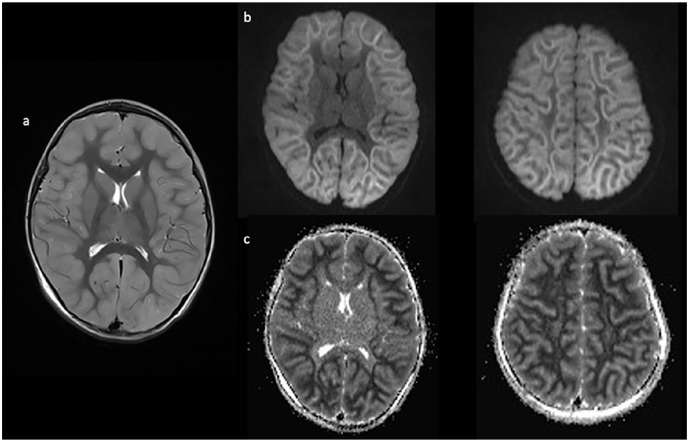
Imaging completed on day 8 of illness shows cortical inflammation on axial T2 (a) with associated diffusion restriction (b) and corresponding ADC changes (c) diffusely in a bright tree appearance.

*Case 2*: A 4-year-old, previously healthy, and developmentally normal Southeast Asian girl presented to hospital on day 3 of a respiratory illness with 2 short (30 seconds) generalized seizures in the context of fever. Her initial head CT was normal, but her liver enzymes were significantly elevated (ALT > 10 000 U/L, aspartate transaminase [AST] 2600 U/L, and international normalised ratio [INR] 4.4). Bilirubin and ammonia were normal, there were no CSF abnormalities, and an infection screen was negative. EEG showed diffuse generalized slowing, high-voltage rhythmic activity, and lack of sleep features. Brain MRI on day 5 was normal, but by day 8, the repeat brain MRI (Figure 2) showed restricted diffusion throughout the entire cortex with the characteristic BTA.

**Figure 3.**
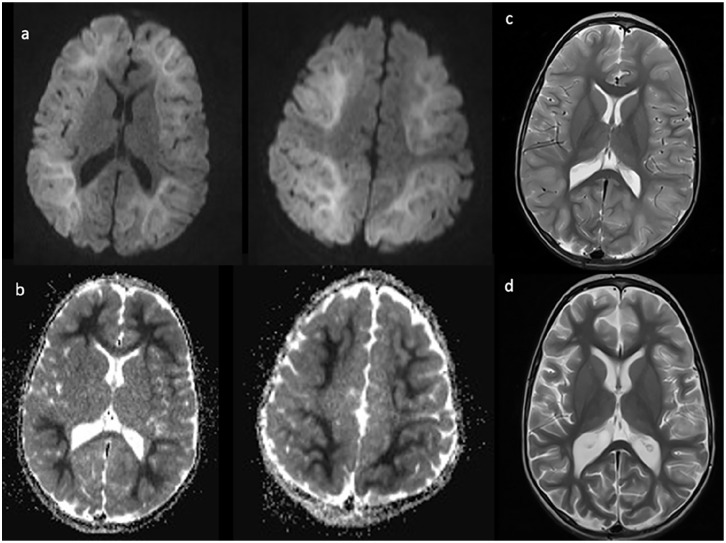
Imaging completed on day 5 of illness shows diffusion restriction (a) and corresponding ADC changes (b) diffusely in a bright tree appearance with corresponding cortical inflammation on axial T2 (c). Repeat axial T2 images (d) 1 month after presentation display diffuse cerebral volume loss and increased cortical T2 signal.

*Case 3*: A 3-year-old, developmentally normal white boy with a history of panhypopituitarism secondary to an ectopic posterior pituitary presented to hospital on day 2 of a febrile respiratory illness with prolonged (>30 minutes) febrile status epilepticus (FSE) and hypoglycemia (lowest measured glucose 1.8 mmol/L). His hypoglycemia was corrected quickly, but multiple antiepileptic medications were needed to control FSE. An initial head CT did not show any abnormalities, and initial blood work including an infectious screen and CSF studies were normal. On day 4, he developed recurrent afebrile focal seizures, and an EEG showed nonspecific diffuse slowing and lack of sleep features. On day 5, a head MRI (Figure 3) showed diffuse restricted diffusion of the white matter with a BTA sparing the primary sensorimotor cortex. His ALT was mildly elevated at 71 U/L.

## Final Diagnosis

Febrile status epilepticus secondary to acute encephalopathy with biphasic seizures and delayed diffusion restriction (AESD).

## Hospital Course

Pertinent investigations and patient demographics can be seen in Table 1.

**Table 1.** Pertinent Investigations and Patient Demographics.

	Case 1	Case 2	Case 3
Age	22 months	4 years	3 years
Ethnicity	White	Southeast Asian	White
Past medical history	Simple febrile seizure at 15 months	Healthy	Ectopic posterior pituitary
Virus	Enterovirus (sputum)	None isolated	Enterovirus (sputum)
Liver enzymes/function	ALT >1200 U/L INR normal Ammonia normal	ALT > 10 000 U/L INR 4.4 Ammonia normal	ALT 71 U/L INR normal Ammonia normal
EEG	Generalized slowing, lack of sleep features	Generalized slowing, lack of sleep features	Generalized slowing, lack of sleep features
MRI	Day 6: Diffusion restriction with a BTA and sparing of the peri-rolandic areas	Day 8: Diffusion restriction in entire cortex and bilateral cerebral hemispheres with a BTA	Day 5: Diffusion restriction with a BTA sparing sensorimotor cortex
Treatment	Steroids Vitamin B6 Temperature regulation	Carnitine	Steroids Plasmapheresis
Outcome	Death	Significant motor and cognitive delays	Significant motor and cognitive delays, PEE

Abbreviations: ALT = alanine transaminase; INR = international normalised ratio; EEG = electroencephalography; BTA = bright tree appearance; MRI = magnetic resonance imaging; PEE = post-encephalopathic epilepsy.

*Case 1*: Aggressive neuroprotective techniques were initiated, but the patient developed autonomic instability and progressive cerebral edema. Pulsed steroids and vitamin B6 were trialed. A brain biopsy showed anoxic-ischemic injury and necrosis. Due to persistent coma and progressive cerebral edema, comfort care was initiated on day 10. Autopsy showed acute/subacute anoxic-ischemic changes bilaterally within the neocortex, hippocampus, and basal ganglia, with cerebellar cortex, brainstem, and spinal cord sparing.

*Case 2*: The patient continued to develop cerebral edema requiring aggressive neuroprotective measures and carnitine was trialed. A repeat MRI on day 16 showed a mild improvement in diffusion restriction. She was discharged with severe motor and cognitive impairment.

*Case 3*: During the acute illness, the patient developed significant developmental regression of language and motor skills. He subsequently received high-dose steroids followed by 5 courses of plasmapheresis, which modestly improved his symptoms. One month after presentation, his brain MRI showed supratentorial cerebral volume loss and diffusely increased cortical T2 signal. Development improved over time, but he continued to show significant language and fine and gross motor skill delays, and he developed post-encephalopathic epilepsy (PEE).

## Discussion

Pediatric encephalopathy is a serious condition with an overall mortality rate of ~5%.^1^ The AESD is well described in Asian countries.^2^ It is the most common pediatric encephalopathy syndrome described in Japan, accounting for 5% to 30% of encephalopathy cases.^3,4^ The median age of presentation is 15 to 19 months of age, with a female predominance and generally low mortality but high morbidity rate.^3,5^ While there are rare case reports internationally, there is only a single case reported in the United States^6^ and no cases reported in Canada. It remains unclear whether the disorder is underrecognized or truly uncommon in North America, but these cases suggest that AESD can occur outside Japan and in children of various ethnicities. Awareness of AESD and early consideration of the diagnosis are critical, as prompt management is the most effective prognostic factor.

The AESD can be distinguished from other encephalopathies by the presence of biphasic seizures and characteristic BTA on diffusion-weighted imaging (DWI)-MRI.^5^ Patients present with prolonged febrile seizures and delayed time to awakening followed by transient improved consciousness for 3 to 7 days, after which consciousness deteriorates and clusters of afebrile seizures develop.^7^ Transaminitis and cerebral edema are commonly seen as the disease progresses.^7^ Early brain MRI is typically normal, but 3 to 14 days after onset, DWI-MRI shows restricted diffusion in the bilateral subcortical white matter described as a BTA pattern and usually centro-occipital sparing.^5,7^ The BTA pattern appears to be a sensitive marker for AESD, although similar patterns can be seen in other diseases. Patients usually survive the acute episode, but neurological sequelae can be serious.^3,5,8^

The early clinical differentiation of AESD and FSE with a prolonged waking time can be challenging. Prolonged seizures at the time of presentation followed by a prolonged time to awakening are useful diagnostic features of AESD. Yokochi et al^4^ found that time to awakening was 11 hours in AESD patients compared with 4 hours in non-AESD patients; however, sedating medications including antiepileptics make the interpretation of prolonged time to awakening challenging. The diagnosis of AESD was made in our patients after the second afebrile biphasic seizures developed, thus delaying treatment. As seen in our patients, elevated liver enzymes, lactate dehydrogenase, ammonia, creatinine and respiratory acidosis have been reported to be more common in AESD than in febrile seizures.^1,4,8,9^ Also, like our patients, diffusely slow EEG with lack of sleep spindles within 48 hours of disease onset has also been considered to be a potential early biomarker of AESD.^7^ While there is still no definitive early biomarker of AESD, each case in our series had some combination of these predictive factors. Also, the most common infectious agents identified in patients with AESD include the influenza virus, HHV-6, enterovirus, and adenovirus,^3,5,7,8^ and indeed enterovirus was identified in 2 of our patients.

The uncertain pathophysiology of AESD contributes to the difficulty in devising optimal management strategies. Glutamate and subsequent neuronal cell death have been proposed to be involved in its pathogenesis,^10^ the hypothesis being that glutamate is released at the time of the first seizure, followed by a gradual increase in glutamine, leading to secondary deterioration and cytotoxic edema.^11^ Magnetic resonance spectroscopy (MRS) studies of children with AESD further support this hypothesis.^10,11^

Inhibition of glutamate release by hypothermia or cooling has shown efficacy in AESD patients^10,12^ and hypothermia has antiepileptic effects.^7^ While there is some evidence for the beneficial effect of early temperature regulation within 6 hours of symptoms onset in AESD,^10,12^ treating patients prior to identifying an etiology may result in many patients being unnecessarily cooled. Yokochi and colleagues^4^ initiated therapeutic hypothermia following the second seizure onset and concluded that as therapeutic hypothermia was associated with a good outcome in 50% of patients, temperature regulation might still be effective even if initiated later in the disease course. The glutamate theory may also explain why immunomodulating treatments that suppress inflammatory cytokines, such as high-dose steroids and IVIg, have little or no effect in AESD, because it is not a disorder of cytokine secretion.

Another proposed pathogenetic mechanism implicates energy failure followed by mitochondrial dysfunction. The AESD patients found to have a lactate peak on MRS, which reflects a shift toward anaerobic glycolysis and an energy supply and demand imbalance, had a worse outcome.^10^ Consistent with this hypothesis, early initiation of vitamins and co-enzymes including vitamin B1, B6 and l-carnitine has been proposed as a therapy and can improve outcome in suspected AESD cases.^13,14^

Early recognition is important, as delay has been shown to have cellular and molecular consequences. Shiihara and colleagues^15^ reported that tau protein, a CSF biomarker of axonal damage, is elevated in children with AESD within the first 2 days of illness, suggesting that waiting to treat until the appearance of biphasic seizures or until BTA changes are observed may be too late to ameliorate pre-existing brain damage.

While the mortality rate for AESD is reported to be low in Japan (<5%), a wide spectrum of adverse neurodevelopmental outcomes have been described.^3,5,8^ In all, 28% to 45% of patients have a good recovery, 40% to 50% experience mild deficits, and 5% to 45% severe sequelae.^3,5,8^ Poor outcomes include motor and/or cognitive impairment as well as refractory epilepsy. PEE has been reported to occur 2 to 39 months after AESD and often involves multiple seizure types which are difficult to control.^2,12^ Here, 1 case resulted in withdrawal of care, whereas the remaining 2 had significant morbidities and 1 developed PEE, which may reflect the relatively late diagnosis of AESD in our practice. Nevertheless, it still remains to be determined whether AESD represents a disease spectrum that impacts severity and outcomes.

## Conclusion

The AESD should be suspected in children of any ancestry presenting with febrile seizures followed by prolonged time to awakening associated with transaminitis and an abnormal EEG. Further research is needed to identify specific biomarkers of AESD to facilitate early management, which might include vitamin B1, vitamin B6, l-carnitine, and temperature regulation.
